# Supplementary material for: Symbiont effectors modulate plant signaling to increase host stress resilience
Source: Plant Physiol. 2026 Sep 8;202(2):kiag664. doi: 10.1093/plphys/kiag664 (PMC13655452; doi:10.1093/plphys/kiag664)
Supplement: Supplementary file 1 [file kiag664_supplementary_data.zip › SupplementaryFigures_resubmission PLPHYS-2025-0056R1.pdf]

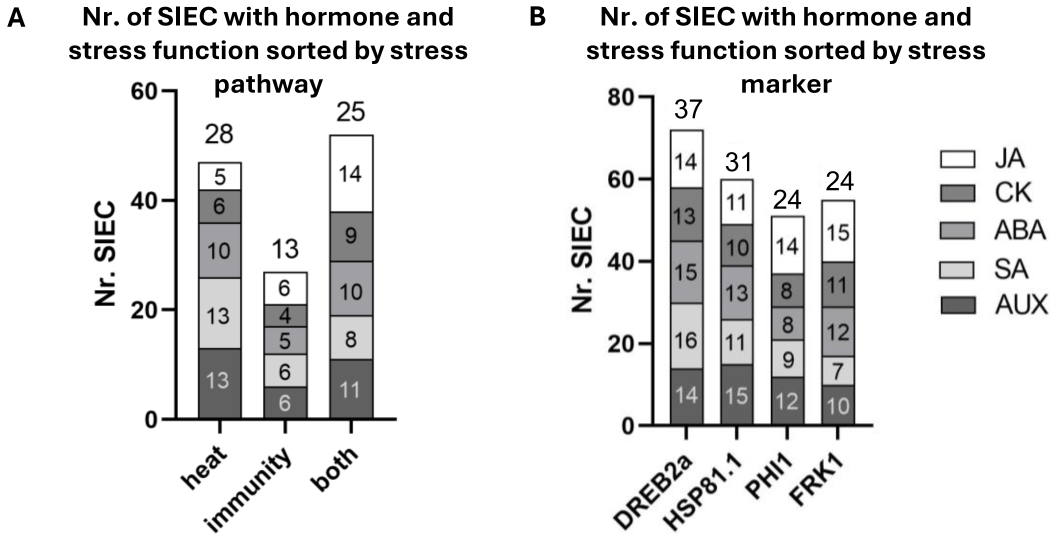

Supplementary Figure 1. Number of SIECs with stress and hormone function.
A Number of SIECs that alter stress marker expression and have additional hormone functions. **B** Number of SIEC that specifically modulate stress signalling and, in addition, hormone pathways. The number above each bar indicates the total number of SIECs which is lower than the sum as individual SIECs can affect multiple hormone and stress pathways. AUX – auxin, CK – cytokinin, ABA – abscisic acid, JA – jasmonic acid, SA – salicylic acid

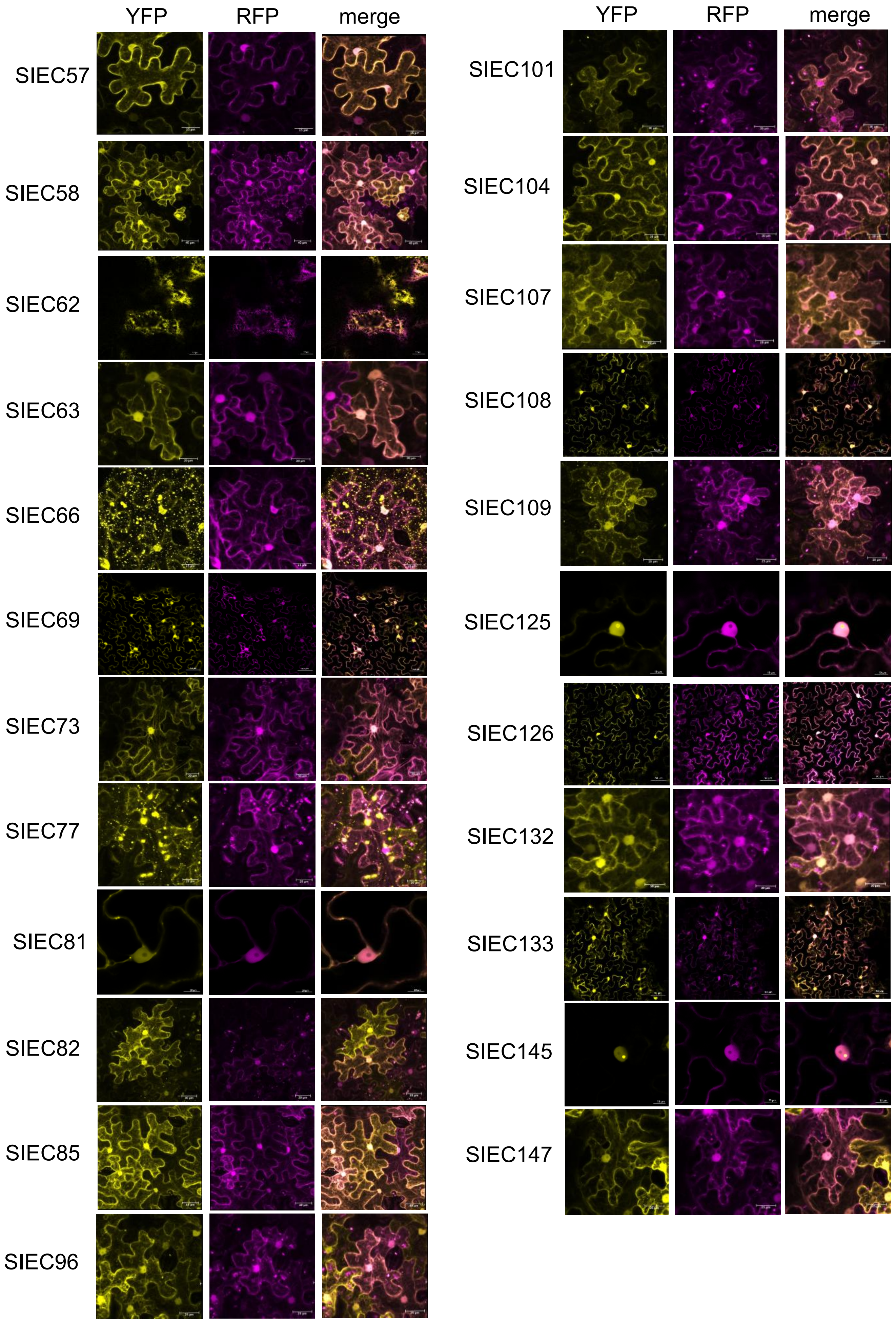

Supplementary Figure 2. Confocal laser scanning microscopy images.

Supplementary Figure 2. Confocal laser scanning microscopy images.

Confocal laser scanning microscopy images of SIEC-YFP fusions (yellow) transiently expressed in *N. benthamiana* epidermal cells. Subcellular markers fused to RFP were co-expressed alongside each effector to denote specific organelles (magenta).

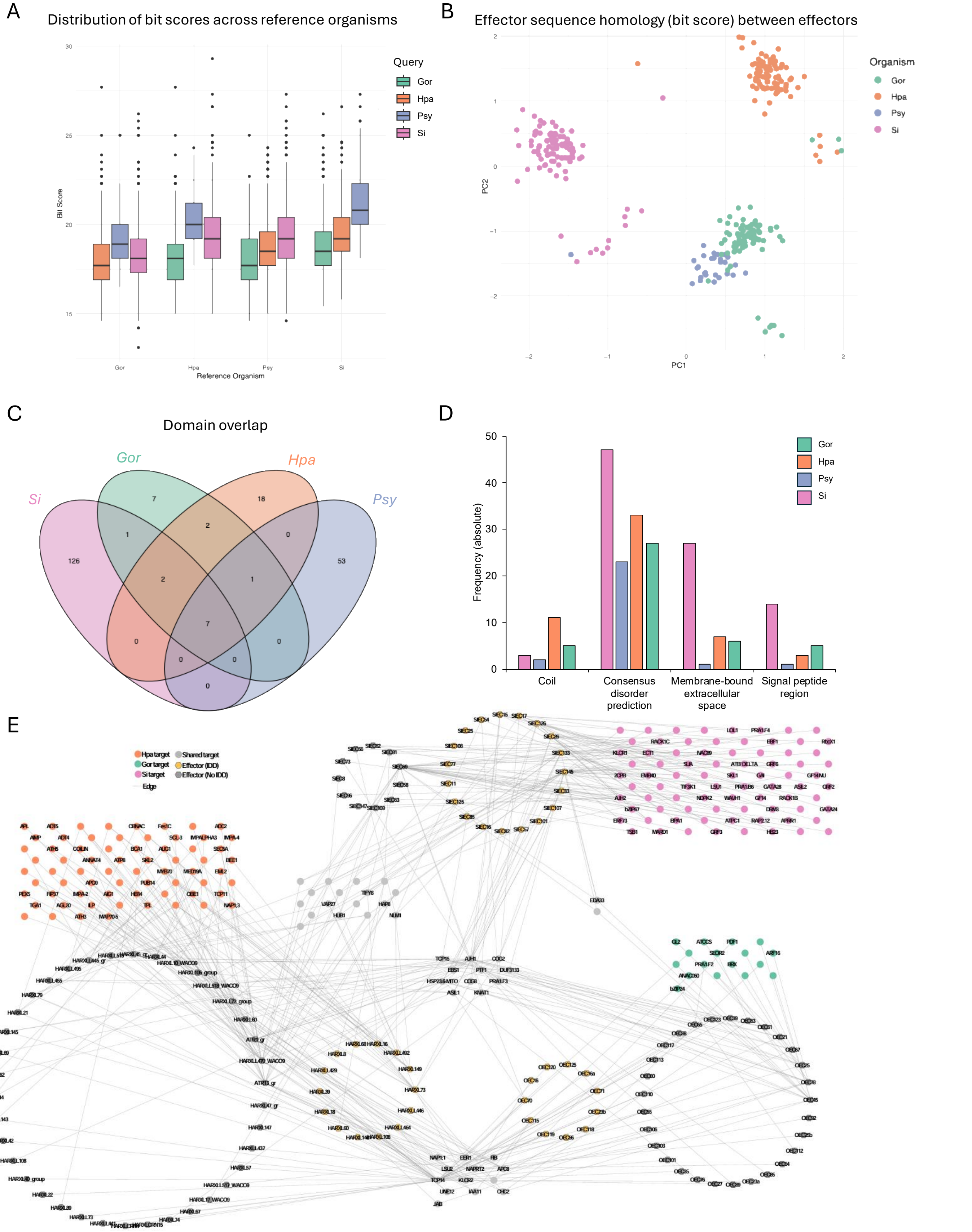

Supplemental figure 3. Sequence and protein domain analysis of microbial effectors

A Box and whisker plots showing Bit score across reciprocal pBLAST analyses between *Si*, *Hpa*, *Psy* and *Gor* effector sequences. **B** PCA describing variance between sequence homology scores for each effector from Si, *Hpa*, *Psy* and *Gor*. **C** Venn diagram showing overlap in protein domains for microbial effectors predicted using EBI InterPro.**D** Absolute frequency of the most prevalent protein domains predicted for microbial effectors. **E** Network graph displaying interactions between effectors of *Si*, *Gor* and *Hpa* within the 12k search space. Effectors (hexagons) are classified based on the presence of an intrinsically disordered domain. Convergent host targets (circles) interact with both IDD and IDD-absent effector proteins.

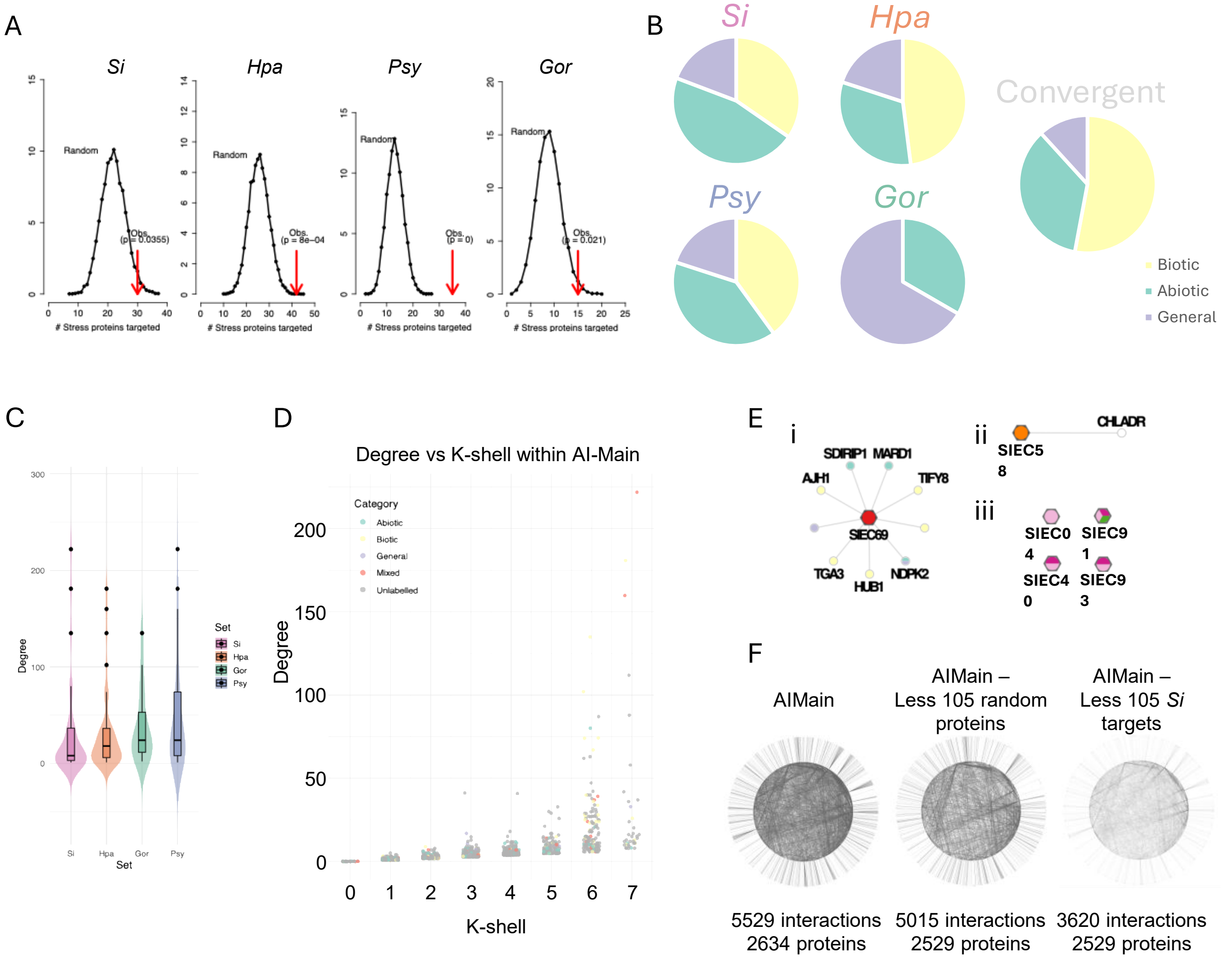

Supplemental figure 4. Effector proteins target stress responsive proteins

A Frequency of stress protein targeting by effectors in random networks (black dots) vs the observed rate of stress protein targeting (red arrows) in respective interactomes for *Si*, *Hpa*, *Psy* and *Gor*. **B** Categorisation of stress associated proteins amongst unique effector targets for *Si*, *Hpa*, *Psy Gor* and convergent targets. Charts comprise every semantic appearance of each stress category, i.e. a protein with a function in both abiotic and biotic stress appears in both sections of the pie chart. **C** Violin plot displaying the degree of effector targets within AI-Main sorted by organism. **D** Distribution of nodes within AI-Main when considering degree vs K-shell. Stress annotated proteins appear within each K-shell with no trend toward lower vs higher degree. **E** Subnetworks displaying interactions of SIECs used in abiotic stress phenotyping; (i) SIEC with multiple stress targets, (ii) SIEC with one stress related target, (iii) SIEC with known stress marker effects with no identified interactors. **F** Network graph displaying the interconnectivity of AI-Main. Removal of *Si* target proteins reduces the total number of interactions by ~2,000, whereas the removal of an equivalent number of random proteins reduces the connectivity by ~500.

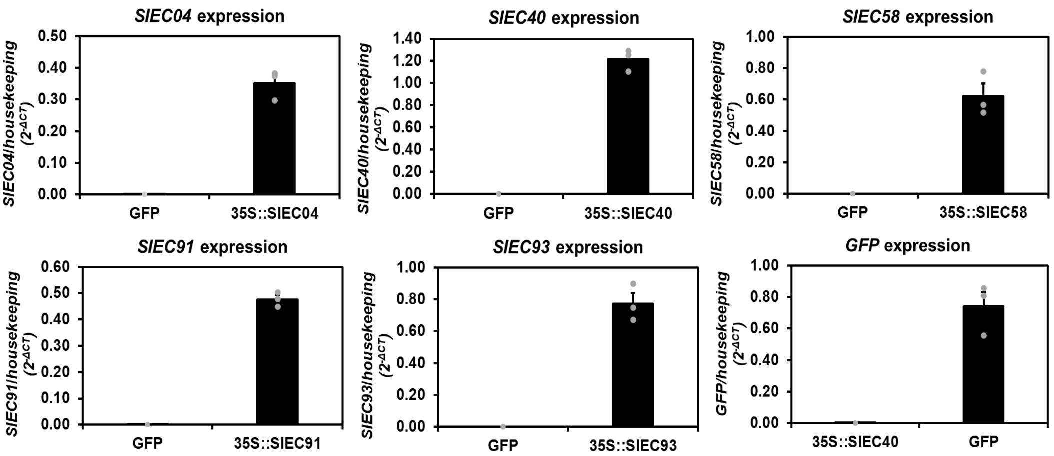

Supplementary Figure 5. qRT-PCR-based quantification of *SlEC* expression in *SlEC*-expressing Arabidopsis seedlings.

RNA of roots of 14-day old seedlings was used to analyse *SlEC* expression by qRT-PCR. *SlEC*-specific primers were used to confirm expression in *p35S::SlEC* lines relative to housekeeping genes *UBQ5* and *EF1α*. GFP-expressing plants served as negative control. Data shown is based on three technical replicates of RNA extracted from a pool of *p35S::SlEC* seedlings.

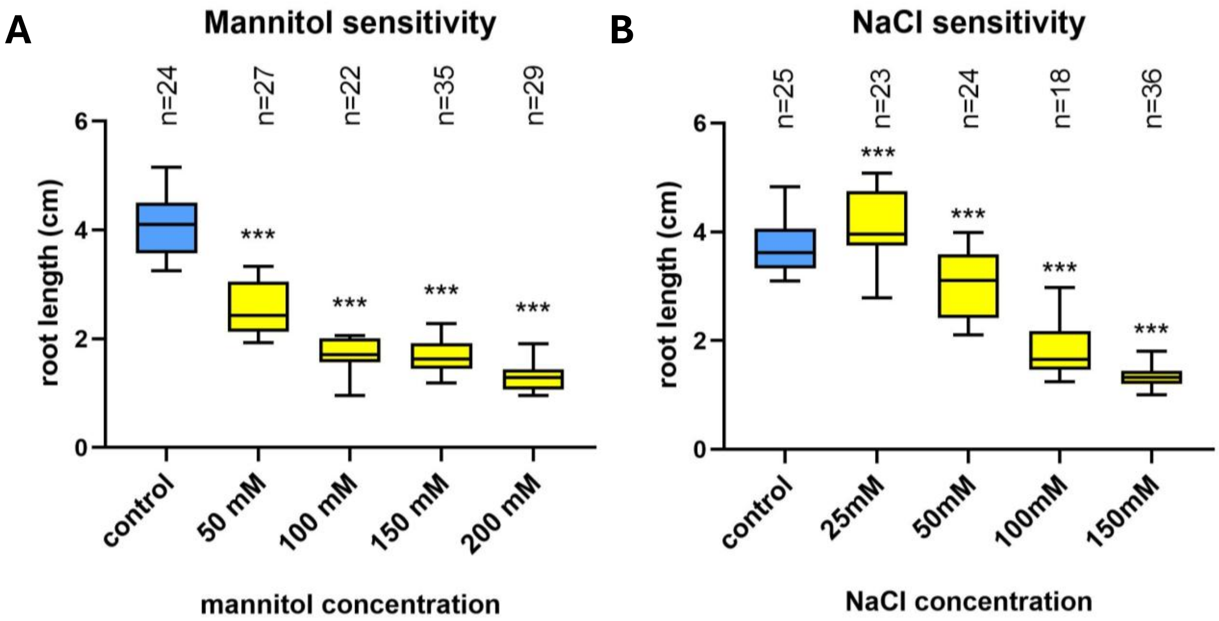

Supplementary Figure 6. Mannitol and NaCl sensitivity assays.

A Effect of ascending concentrations of mannitol and **B** NaCl on primary root length of 14-day old seedlings. Root length of control plants is indicated in blue. (***) $p < 0.001$; statistical significance was evaluated by ANOVA. Sample size (n) is indicated above each box.

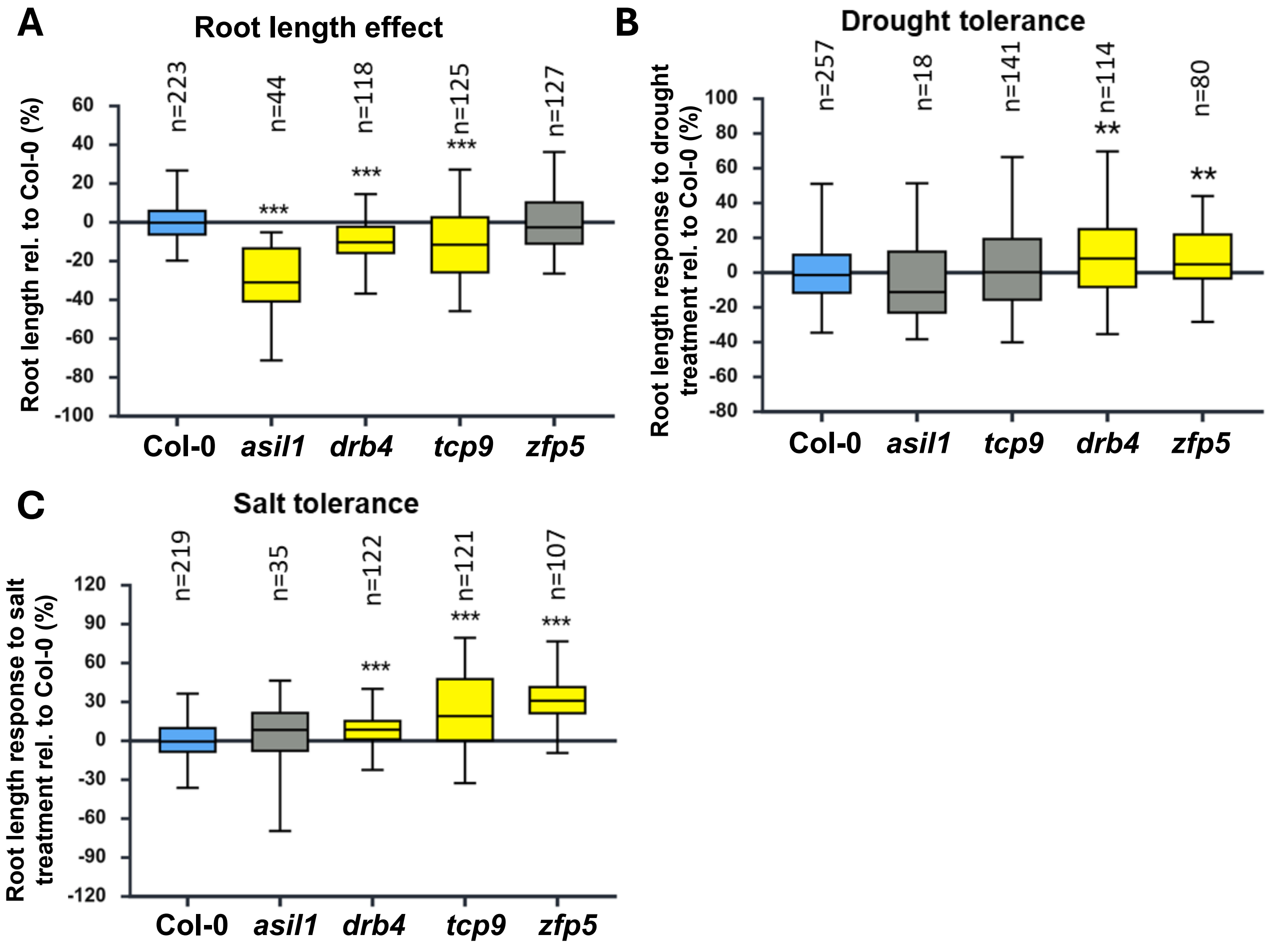

Supplementary Figure 7. Phenotyping of target knockout lines.
A Primary root length, **B** relative drought stress and **C** relative salt stress response of primary root length of 14-day old seedlings relative to Col-0 (%). Root length of control plants is indicated in blue. (***) $p < 0.001$; statistical significance was evaluated by ANOVA. Sample size (n) is indicated above each box.
